# Highly Thermal Conductive Nanocomposites

**DOI:** 10.3390/nano13091443

**Published:** 2023-04-23

**Authors:** Xiaoliang Zeng

**Affiliations:** Shenzhen Institute of Advanced Electronic Materials, Shenzhen Institutes of Advanced Technology, Chinese Academy of Sciences, Shenzhen 518055, China; xl.zeng@siat.ac.cn

The Special Issue of *Nanomaterials*, “Highly Thermal Conductive Nanocomposites”, focuses on the application of different types of thermal conductivity nanocomposites in thermal management. The wide variety of nanocomposites covered by the eight articles published here is proof of the growing attention that the use of nanocomposites in thermal management has received in recent years.

This Special Issue is intended to provide readers with a compilation of cutting-edge research regarding the synthesis, development, and characterization of highly thermal conductive nanocomposites as a guide for new students as well as established researchers in this field.

Thermal conductive nanocomposites with randomly distributed nano-filler are presented in this Special Issue. For example, Fatema Tarannum et al. fabricated polyetherimide-graphene and epoxy-graphene nanocomposites via solvent casting technique [1]. A thermal conductivity of 6.6 W m^−1^ K^−1^ is achieved for a 10 wt% graphene composition sample, representing an enhancement of ~2770% over pristine polyetherimide (~0.23 W m^−1^ K^−1^). This enhancement in thermal conductivity is shown to be due to a network of continuous graphene sheets over long−length scales, resulting in low thermal contact resistance at bends/turns due to the graphene sheets being covalently bonded at such junctions. Solvent casting offers the advantage of preserving the porous structure of expanded graphite in the composite, resulting in the above highly thermally conductive interpenetrating network of graphene and polymer. Solvent casting also does not break down the expanded graphite particles due to minimal forces involved, allowing for efficient heat transfer over long−length scales, and further enhancing overall composite thermal conductivity. Juncheng Xia and coworkers prepared a fishbone-like silicon carbide (SiC) material and prepared silicon carbide/polyvinylidene fluoride (SiC/PVDF) thermal conductive nanocomposites by combining it with polyvinylidene fluoride (PVDF) [2]. The thermal conductivity of the prepared nanocomposites is 0.92 W m^−1^ K^−1^ at 70 wt% SiC content, which provides a new idea for its application in the field of electronic packaging. The results show that using the filler with a new structure to construct thermal conductivity networks is an effective way to improve the thermal conductivity of PVDF. Yuge Ouyang et al. reported a thermal conductive polymer composite composed of silicone rubber (SR) and branched Al_2_O_3_ (B-Al_2_O_3_) [3]. Due to the unique two-dimensional branching structure of B-Al_2_O_3_, the maximum thermal conductivity of the polymer nanocomposite with 70 wt% filler is 1.242 W m^−1^ K^−1^, which is 521% higher than that of the pure matrix. In addition, B-Al_2_O_3_ fillers are well dispersed (no large agglomerates) and form a strong interfacial adhesion with the matrix. Therefore, the thermal decomposition temperature, residual mass, tensile strength, modulus, and modulus of the toughness of composites are significantly improved simultaneously. Excellent dielectric breakdown strength is also important to some applications of thermal conductive nanocomposites. Zhengdong Wang and coworkers prepared a new type of AlN/epoxy sandwich composite, which showed significantly enhanced dielectric breakdown strength and thermal conductivity [4]. The most optimized sandwich composite, with an outer layer thickness of 120 μm and an inner layer thickness of 60 μm (abbreviated as 120–60) exhibits a high through-plane thermal conductivity of 0.754 W m^−1^ K^−1^ (4.1 times of epoxy) and has a dielectric breakdown strength of 69.7 kV mm^−1^, 8.1% higher compared to that of epoxy. The sandwich composites also have higher in-plane thermal conductivity (1.88 W m^−1^ K^−1^ for 120–60) based on the novel parallel models. The sandwich composites with desirable thermal and electrical properties are very promising for application in power electronic devices and power equipment.

Thermal conductive nanocomposites with a directional arrangement of fillers are also presented in this Special Issue. Mingming Yi et al. prepared polyethylene/boron nitride nanoplates (PE/BNNPs) nanocomposites for thermal management in devices like light-emitting diodes [5]. The thermal conductivity of the prepared PE/BNNPs nanocomposites is 0.47 W m^−1^ K^−1^, which gets a 14.6% improvement compared to pure polyethylene film. The fracture stress was also highly enhanced, with an increase of 148.44% compared to pure polyethylene film. Moreover, the addition of BNNPs in PE does not highly reduce its good transmittance, which is preferred for thermal management in devices like light-emitting diodes. For high in-plane thermal conductivity, Cenkai Xu and coworkers report a new strategy for preparing mechanically strong and thermally conductive composite films by combining aramid nanofibers (ANFs) with graphene oxide (GO) and edge-hydroxylated boron nitride nanosheet (BNNS-OH) via a vacuum-assisted filtration and hot-pressing technique [6]. The obtained ANF/GO/BNNS film exhibits an ultrahigh in-plane thermal conductivity of 33.4 W m^−1^ K^−1^ at the loading of 10 wt.% GO and 50 wt.% BNNS-OH, which is 2080% higher than that of pure ANF film. The exceptional thermal conductivity results from the biomimetic nacreous “brick-and-mortar” layered structure of the composite film, in which favorable contacting and overlapping between the BNNS-OH and GO is generated, resulting in tightly packed thermal conduction networks. In addition, an outstanding tensile strength of 93.3 MPa is achieved for the composite film, owing to the special biomimetic nacreous structure as well as the strong π−π interactions and extensive hydrogen bonding between the GO and ANFs framework. Meanwhile, the obtained composite film displays excellent thermostability (*T_d_* = 555 °C, *T_g_* > 400 °C) and electrical insulation (4.2 × 10^14^ Ω cm). In the field of thermal interface materials (TIMs), Yuanzhou Chen et al. fabricated a series of flexible fiber membranes (TMMFM) that is highly thermally conductive based on thermoplastic polyurethane (TPU) and acidified multiwalled carbon nanotubes (a-MWCNTs) via the electrospinning and ultrasonic anchoring method [7]. Due to the orientation of a-MWCNTs, with the filler addition of 10 wt%, the horizontal direction (λ_∥_) and vertical direction (λ_⊥_) thermal conductivity value of TMMFM-5 was 3.60 W m^−1^ K^−1^ and 1.79 W m^−1^ K^−1^, respectively, being 18 times and 10 times higher compared to pure TPU fiber membranes. Furthermore, the TMMFM maintained favorable flexibility of the TPU matrix because the small amount of a-MWCNTs only slightly hinders the mobility of the TPU molecular chain. The performance of the obtained TMMFM unveils their potential as a promising choice of flexible TIMs.

Our Special Issue also covers high-quality review articles. Humaira Yasmin et al. made a review about the influence of several parameters on the thermal conductivity of metal oil nanofluids, and the mechanisms/physics behind thermal conductivity enhancement and techniques for thermal conductivity measurement have been discussed [8]. Thermal conductivity and thermal conductivity enhancements of metal oxide nanofluids are presented and discussed herein. The influence of several parameters (temperature, volume/weight concentration, nano-size, sonication, shape, surfactants, base fluids, alignment, thermal conductivity measurement techniques, and mixing ratio (for hybrid nanofluid)) on the thermal conductivity of metal oil nanofluids have been reviewed. This paper serves as a frontier in the review of the effect of alignment, electric field, and green nanofluid on thermal conductivity. In addition, the mechanisms/physics behind thermal conductivity enhancement and techniques for thermal conductivity measurement have been discussed. Results show that the thermal conductivity enhancement of metal oxide nanofluids is affected by the aforementioned parameters with temperature and nanoparticle concentration contributing the most.

In summary, this Special Issue presents several examples of the latest advancements in thermal conductive nanocomposites. We hope the readers will enjoy reading these articles and find them useful for their research.
